# Isochromosome 17q in Chronic Lymphocytic Leukemia

**DOI:** 10.1155/2015/489592

**Published:** 2015-11-30

**Authors:** Eyad Alhourani, Martina Rincic, Joana B. Melo, Isabel M. Carreira, Anita Glaser, Beate Pohle, Cordula Schlie, Thomas Liehr

**Affiliations:** ^1^Jena University Hospital, Friedrich Schiller University, Institute of Human Genetics, Kollegiengasse 10, 07743 Jena, Germany; ^2^Croatian Institute of Brain Research, Salata 12, 1000 Zagreb, Croatia; ^3^Laboratory of Cytogenetics and Genomics, Faculty of Medicine, University of Coimbra, Polo Ciências da Saúde, 3000-548 Coimbra, Portugal; ^4^Centro de Investigação em Meio Ambiente, Genética e Oncobiologia (CIMAGO), Polo Ciências da Saúde, 3000-548 Coimbra, Portugal

## Abstract

In chronic lymphocytic leukemia (CLL), presence of acquired cytogenetic abnormalities may help to estimate prognosis. However, deletion of* TP53* gene, which is associated with an aggressive course of the disease and poor prognosis along with a lack of response to treatment, is one of the alterations which may escape cytogenetic diagnoses in CLL. Thus, other techniques have emerged such as interphase fluorescence* in situ* hybridization (iFISH). Deletion of* TP53* may but must not go together with the formation of an isochromosome i(17q); surprisingly this subgroup of patients was not in the focus of CLL studies yet. This study was about if presence of i(17q) could be indicative for a new subgroup in CLL with more adverse prognosis. As a result,* TP53* deletion was detected in 18 out of 150 (12%) here studied CLL cases. Six of those cases (~33%) had the* TP53* deletion accompanied by an i(17q). Interestingly, the cases with i(17q) showed a tendency towards more associated chromosomal aberrations. These findings may be the bases for follow-up studies in CLL patients with* TP53* deletion with and without i(17q); it may be suggested that the i(17q) presents an even more adverse prognostic marker than* TP53* deletion alone.

## 1. Introduction

Chronic lymphocytic leukemia (CLL) is a relatively frequently observable acquired disease in men and women of >50 years of age [1]. Also CLL is a heterogeneous malignancy, as the survival of CLL patients can be in the range of months to decades according to the underlying genetic abnormalities [2]. The most frequent cytogenetic aberrations in CLL are involving chromosomal subbands 13q14 (50–60%), 14q32 (12–15%), 11q22 (10–20%), and 17p13 (5–10%) as well as trisomy 12 (15–25%); each group has different prognoses and survival rates [1, 3]. Deletion of* TP53* gene, which is located in the short arm of chromosome 17 towards the telomeric region in 17p13.1, is associated with poor prognosis and lack of response to fludarabine-based regimens.

*TP53* deletion in CLL can be associated with isochromosome formation of the long arm of one chromosome 17 leading at the same time to partial monosomy 17p and partial trisomy 17q. In general, isochromosome i(17q) is the most frequently observed isochromosome in hematological malignancies and it can be present as primary or secondary aberration; that is, it may play roles during development as well as progression of the malignancy. Presence of i(17q) as a sole abnormality is associated with a high risk of progression and an aggressive clinical course, but i(17q) can also be found as part of a complex karyotype [4–6]. In solid tumors, i(17q) is reported predominantly in medulloblastoma [7], there often associated with c-myc amplification [8].

Overall, detection of acquired chromosomal abnormalities such as i(17q) just based on GTG-banding may be limited due to low mitotic potential of CLL bone marrow cells. Thus, nowadays other techniques are applied to overcome this problem, by name interphase fluorescence* in situ* hybridization (iFISH), multiplex ligation dependent probe amplification (MLPA), and array-comparative genomic hybridization (aCGH) [2, 9, 10]. Here we studied 150 CLL samples and concentrated on the questions (i) if i(17q) can be detected reliably by MLPA and (ii) if i(17q) presence in patients with* TP53* deletion is associated with more complex cytogenetic aberrations. An association with the clinical outcome would have been favorable as well; unfortunately this was not possible due to lack of necessary clinical data.

## 2. Material and Methods

### 2.1. Patients and Sample Preparation

The present study included 150 CLL patients, which were diagnosed according to standard criteria [11]. The samples were obtained under informed consent of the corresponding patients and according to institutional ethical committee guidelines (Ethical Committee of the Friedrich Schiller University Jena).

DNA was extracted from lymphocytes of 85 CLL cases by a commercial kit (Qiagen) according to manufacturer's instructions. DNA was derived from different sources: 2 samples from heparinized bone marrow, 8 samples from heparinized blood, and 75 samples from cytogenetically prepared cells fixed in methanol/acetic acid (3 : 1)—48 of them derived from bone marrow and 27 from blood. Details on the studied patients can be found in the paper by Alhourani et al. (2014): the 10 here in more detail studied patients with* TP53* deletion (Table 1) were cases 61 (now 1), 1 (now 2), 17 (now 3), 19 (now 4), 12 (now 5), 38 (now 6), 18 (now 7), 16 (now 8), 39 (now 9), and 13 (now 10) from Alhourani et al. (2014) [1]. In the previous study, no special attention was given to the here treated i(17q) problem, and additional studies, esp. FISH experiments, and reinterpretation of MLPA and aCGH data were performed here.

For further investigation of i(17q) status, additional 65 CLL patients were included in this study with special focus on 8 cases (86 to 93) with* TP53* (Table 1).

### 2.2. GTG-Banding and Interphase-Directed Fluorescence* In Situ* Hybridization (iFISH) Analysis

GTG-banding and iFISH analyses were done as previously reported [1].

For iFISH, the following probes were used:

- Abbott/Vysis (Wiesbaden, Germany): LSI p53/LSI ATM (in 17p13.1 and 11q22.3), LSI D13S319/LSI 13q34/CEP 12 (in 13q14.3, 13q34, and 12p11.1-q11.1), LSI IGH dual color, break-apart probe (in 14q32.33), LSI SMS Region SpectrumOrange/LSI RARA SpectrumGreen (in 17p11.2 and 17q12-21), CEP 17 (D17Z1 in 17p11.1-q11.1), TelVysion 17p (282M16/SP6), and TelVysion 17q (D17S928).
- From Zytovision (Bremerhaven, Germany): ZytoLight SPEC CMYC/CEN 8 Dual Color (8q24.21 and 8p11.1-q11.1).
- BACPAC Resources Center (Oakland, USA): RP11-318A15 in 17q25.1 (gene* UNC13D*) and RP11-94L15 in 17q12 (gene* IKZF3*).

For each iFISH analysis, 100–200 interphase nuclei were examined per case and probe.

### 2.3. Multiplex Ligation Dependent Probe Amplification (MLPA) Analysis

Multiplex ligation dependent probe amplification (MLPA) was performed on 85 CLL cases using SALSA MLPA probemix P377-A1 for Hematological Malignancies Kit from (MRC-Holland, Amsterdam, Netherlands).

The P377-A1 probemix kit contains 52 probes for overall 37 genes;* TP53* which is located on the short arm of chromosome 17 is covered by 4 probes; likewise* UNC13D* and* IKZF3* on q arm were covered by one probe for each of them [1].

### 2.4. High Resolution Array-Comparative Genomic Hybridization (aCGH)

High resolution array-comparative genomic hybridization (aCGH) was performed using Agilent SurePrint G3 Human Genome microarray 180 K (Agilent Technologies, Santa Clara, CA, USA) as previously reported [12].

## 3. Results

Deletion of* TP53* has been detected in 9/85 cases by MLPA. Besides a screening for* TP53* deletion was done by iFISH in all of the studied 85 CLL cases to detect mosaic cases with low percentage of aberrant cells as well. Accordingly,* TP53* deletion was detected in one additional CLL case, being present there in only 11.5% of the studied cells (case 10).

**Figure 1:**
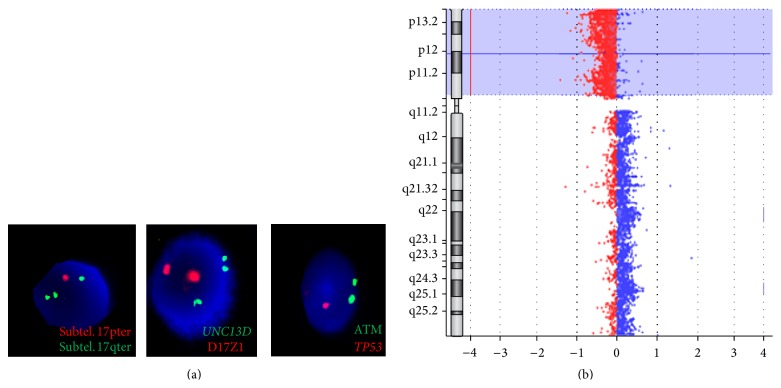
(a) Isochromosome 17q was detected initially by iFISH in this case; representative examples for heterozygote deletions of* TP53* and #17 subtelomeric region 17p (subtel. 17pter) besides three signals for* UNC13D* and subtel. 17qter. Only 2 signals for centromere of chromosome 17 (D17Z1) and ATM gene on chromosome 11 were detected. (b) aCGH showed deletion of short arm and gain of long arm of chromosome 17 in case 3.

The overall detected 10 cases with* TP53* deletion (Table 2) were further studied by iFISH using probes* IKZF3* in 17q12,* UNC13D* in 17q25.1, and subtelomeric probes (17pter and 17qter; Figure 1(a)); furthermore iFISH-probes for the most frequent aberrations in CLL and, in part, aCGH (case 3; Figure 1(b)) have been applied in those cases, as specified by Alhourani et al. (2014). So, overall 3/85 (~3.5%) of here studied CLL cases had the loss of* TP53* due to formation of an i(17q) which is equal to 30% of these patients.

Further 8 cases with* TP53* deletion were found in additional 65 studied CLL patients by iFISH-probe. Here, subtelomeric (17pter and 17qter) probes were applied to identify the three among them cases with i(17q). A probe for 17p11.2 and 17q12 confirmed the isochromosome status in those cases (Table 3).

In the here studied cases with i(17q), this alteration was accompanied by additional chromosomal aberrations (Table 4). For all of them, amplification of c-myc was excluded. While in the first 85 CLL patients, cases 1 and 2 were accompanied by five additional acquired chromosomal rearrangements and case 3 had only one additional change. In cases 1 and 2, at least one of these additional changes was correlated with an adverse prognosis; in case 3 the del(13) is considered to be a favorable prognostic factor. Cases  4–10, which showed just deletion of* TP53* without isochromosome formation, had either no further aberrations (cases 4 and 9) or just one additional chromosomal alteration associated with good prognosis (cases 5, 7, and 10). Only case 8 showed two additional chromosomal alterations with known adverse prognostic meaning.

Among the 8 cases with* TP53* deletion studied only by iFISH, three cases revealed i(17q). While case 88 showed 8 additional chromosomal rearrangements (two of them were associated with good prognosis), the other two cases, 86 and 87, had only one additional chromosomal alteration. The remaining 5 cases with* TP53* deletion and no i(17q) were associated with one additional chromosomal aberration with good prognosis (cases 89 and 90), or no additional chromosomal changes (cases 91, 92, and 93) (Table 1).

## 4. Discussion

Generally, isochromosome formation is characterized by the loss of the entire short arm with subsequent duplication of the entire long arm, resulting in two homologous arms attached to a single centromere as mirror images [4, 7, 13]. There are two hypotheses to explain the formation of isochromosome, either by transverse instead of longitudinal division of the centromere or by chromatid exchange involving two homologous chromosomes. The rate of the appearance of isochromosomes is different among the various types of tumors, with the highest occurrence in germ cell neoplasms (60%) and the lowest in chronic myeloproliferative disorders (2.3%) [14].

Surprisingly, although i(17q) appeared in 6/150 (4%) here studied CLL patients, that is, and 6/18 (~33%) of CLL patients with a deletion of* TP53*, this chromosomal aberration has not been studied in detail yet in this patient group. Still, there is one study including 2 CLL patients with i(17q) which showed that such isochromosome most likely forms due to clustered breakpoints in 17q11 and is not associated with* TP53* mutations of the intact chromosome 17 [4]. In 2006, i(17q) was found to be present in 4/16 (25%) CLL patients with* TP53* gene loss [15]; that is, the here reported frequency is within the same range. However, the initial finding of an i(17q) in 2/21 (9.5%) CLL cases seems to be overestimated due to small sample size [16].

Even though here only 6 cases with i(17q) could be studied, the results summarized in Table 4 show a clear tendency: cases with i(17q) are associated with more aberrations compared to those which have just deletion of* TP53*. Cases  3 and 8 do not exactly fit into this suggestion. However, case 3 had only 25% of the cells with an i(17q) indicating an early phase of the disease; in case 8 cytogenetics provided a hint on an ongoing karyotypic evolution and already advanced stage of the disease.

Whereas Both Baliakas et al. and Rigolin et al. reported that complex karyotype predicts a worse overall survival, also Baliakas et al. demonstrated that complex karyotype is identified as an independent prognostic factor for shorter time-to-first-treatment [17, 18].

Furthermore, Thompson et al. showed that relapsed/refractory CLL patients who reveal del(17p) and complex karyotype have shorter overall survival than those with only del(17p) [19].

Due to lack of clinical data, the clinical impact of i(17q) could not be followed up, but in spite of that the present study gives first hints that i(17q) presence may be an indicator for more aggressive course of CLL disease than just* TP53* deletion without i(17q) formation. Similar findings were reported for other hematological neoplasia, like acute lymphocytic leukemia [20], acute promyelocytic leukemia [21], chronic myeloid leukemia [5], or other myeloid leukemia [22–24].

**Figure 2:**
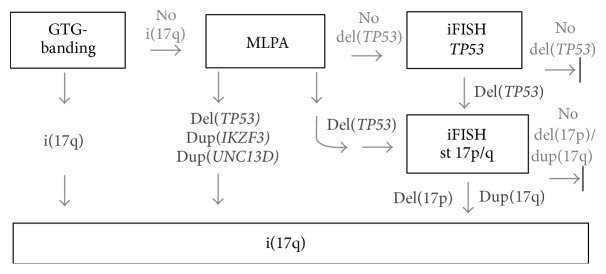
Here a scheme for the suggested procedures how to delineate an i(17q), if cytogenetics, MLPA, and iFISH are available.

As previously outlined by us and others, MLPA is a quick and inexpensive screening tool for CLL diagnostics [1, 25]. However, its inability to detect low level mosaics needs to be considered and thus a diagnostic scheme combining cytogenetics, iFISH, and MLPA needs to be considered for reliable testing of CLL cases in diagnostics [1]. Thus, in Figure 2 we suggest a scheme of how to detect i(17q) reliably.

In conclusion, i(17q) presence in CLL cases with* TP53* deletion should be considered as a potentially adverse marker for more aggressive course of the disease than monosomy of 17p13.1 alone; it needs to be kept in mind that MLPA alone may be not sufficient to pick up all corresponding cases and a combination with iFISH may be considered additionally.

## Figures and Tables

**Table 1:** Gender, age, and cytogenetic results of the 18 studied CLL cases which showed deletion of *TP53* gene.

Case number	Gender	Age [y]	DNA extracted from	Banding cytogenetics
1	F	74	bm	46,XX,i(17)(q10)[1]/ 46,XX,+12,i(17)(q10),-21[9]/ 46,XX,t(3;?)(q2?9;?)[4],-7[4],+12[4],i(17)(q10)[4][cp4]/ 46,XX[4]

2	M	83	bm	47,XY,-11,+12,+mar[cp3]/ 47,XY,del(5)(p1?3),-11,+12,-17,+mar1,+mar2[cp6]/ 46,XY[9]

3	M	72	bm	46,XY

4	F	71	b	46,XX

5	F	50	b	n.a.

6	M	65	bm	46,XY,?t(3;?)(p21;?),der(17)t(17;?),-18,+mar[cp7]/ 46,XY[9]

7	F	66	b	n.a.

8	M	73	bm	46~47,XY,del(11)(q22q2?3),der(17)t(17;?)(p11.2;?)[cp5]/ 45~46,XY,del(11)(q22q2?3),del(17)(p11.2)[cp4]/ 43~46,XY,del(11)(q22q2?3)[cp2]/ 46,XY[7]

9	F	74	B	46,XX

10	F	90	b	n.a.

86	M	74	n.a.	45~46,XY,i(17)(q10)[cp4]/ 45,X,-Y[2]/ 46,XY[14]

87	F	76	n.a.	46,XX,?t(6;19)(p22;p13),del(17)(p?11.2)[1] 46,XX[16]

88	M	65	n.a.	46,XY,t(10;13)(q2?2;q1?3)[10] 46,XY,i(18)(q10)[1] 45,XY,?del(6)(?q21),-17[1] 46,XY,-17,+mar[1] 44,XY,-11,-17[1] 46,XY,-4,-21,+2mar[1] 46,XY[5]

89	F	68	n.a.	n.a.

90	F	63	n.a.	n.a.

91	F	79	n.a.	n.a.

92	M	61	n.a.	n.a.

93	F	75	n.a.	n.a.

b = cell pellet in Carnoy's fixative from blood; bm = cell pellet in Carnoy's fixative from bone marrow; F = female; M = male; n.a. = not available; B = native peripheral blood.

**Table 2:** Summary of MLPA and iFISH results of *IKZF3*- and *UNC13D*-gene specific probes and subtelomeric probes for chromosome 17 in 10 CLL cases with *TP53* deletion in the first group.

Case number	*TP53* [%]		*UNC13D* [%]		*IKZF3* [%]		iFISH [%]	
	MLPA	iFISH	MLPA	iFISH	MLPA	iFISH	*Subtel. pter*	*Subtel. qter*
1	d	d [95]	a	a [90]	a	a [90]	d [90]	a [90]
2	d	d [40]	a	a [40]	a	a [40]	d [40]	a [40]
3	d	d [40]	n	a [25]	n	a [25]	d [25]	a [25]
4	d	d [36]	n	n	n	n	n	n
5	d	d [21]	n	n	n	n	n	n
6	d	d [89]	n	n	n	n	n	n
7	d	d [19]	n	n	n	n	n	n
8	d	d [86]	n	n	n	n	n	n
9	d	d [77]	n	n	n	n	n	n
10	n	d [11,5]	n	n	n	n	n	n
11 to 85	n	n	n	n.a.	n	n.a.	n.a.	n.a.

n = no aberration, d = deletion, a = amplification, n.a. = not tested, and [] = percentage of cells with aberration.

**Table 3:** Summary of iFISH results using SMS and RARA gene specific probes and subtelomeric probes for chromosome 17 in 8 CLL cases with *TP53* deletion in CLL cases only studied by iFISH and not by MLPA.

Case number	iFISH [%]	iFISH [%]		iFISH [%]	
	*TP53*	*Subtel. pter*	*Subtel. qter*	SMS	RARA
86	d [77]	d [77]	a [77]	d [77]	a [77]
87	d [77]	d [77]	a [77]	d [77]	a [77]
88	d [80]	d [80]	a [80]	d [80]	a [80]
89	d [69]	n	n	n.a	n.a
90	d [28]	n	n	n.a	n.a
91	d [75]	n	n	n.a	n.a
92	d [89]	n	n	n.a	n.a
93	d [95.5]	n	n	n.a	n.a
94 to 150	n	n.a	n.a	n.a	n.a

n = no aberration, d = deletion, a = amplification, n.a. = not tested, and [] = percentage of cells with aberration.

**Table 4:** All 18 CLL cases which revealed *TP53* deletion are listed showing the additionally detected chromosomal aberrations and their clinical impact (1).

Case number	Additional aberrations not listing #17 aberrations [%]	Prognosis
1	t(3;?)(q2?9;?)[22]	n.a.
	-7[22]	Adverse
	+12[78]	Intermediate
	del(14)(q32q32)[94]	Good
	-21[50]	n.a.

2	del(5)(p1?3)[33]	n.a.
	del(11)(q22.3q22.3)[30]	Adverse
	+12[70]	Intermediate
	del(13)(q14.3q14.3)[30]	Good
	rea(14)(q32.33)[28] -> ?+14	Adverse

3	del(13)(q14.3q14.3)[20]	Good

4	None detected	Intermediate

5	del(13)(q14.2q14.2)[52]	Good
	del(13)(q14.2q14.2)x2[38]	
	del(13)(q14.3q14.3)[34]	

6	t(3;?)(p21;?)[44]	n.a.
	-18,+mar[44]	n.a.

7	del(13)(q14.3q14.3)[90.5]	Good

8	amp(8)(q24.21q24.21)[21]	Adverse
	del(11)(q22.3q22.3)[11]	Adverse

9	None detected	Intermediate

10	del(13)(q14.3q14.3)x2[98.5]	Good

86	-Y	n.a.

87	?t(6;19)(p22;p13),del(17)(p?11.2)	Adverse

88	t(10;13)(q2?2;q1?3)	Advesrse
	i(18)(q10)	n.a.
	?del(6)(?q21),-17	n.a.
	-17,+mar	n.a.
	-11,-17	n.a.
	-4,-21,+2mar	n.a.
	del(13)(q14.3q14.3)[57]	Good
	del(14)(q32q32)[75]	Good

89	del(13)(q14.2q14.2)[50]	Good
	del(13)(q14.2q14.2)x2[7]	

90	del(14)(q32q32)[36]	Good

91	None detected	Intermediate

92	None detected	Intermediate

93	None detected	Intermediate

n.a. = not available.
